# Association between ambient air pollution and perceived stress in pregnant women

**DOI:** 10.1038/s41598-021-02845-4

**Published:** 2021-12-06

**Authors:** Dirga Kumar Lamichhane, Dal-Young Jung, Yee-Jin Shin, Kyung-Sook Lee, So-Yeon Lee, Kangmo Ahn, Kyung Won Kim, Youn Ho Shin, Dong In Suh, Soo-Jong Hong, Hwan-Cheol Kim

**Affiliations:** 1 Department of Occupational and Environmental Medicine, Inha University School of Medicine, Incheon, Republic of Korea; 2 Department of Psychiatry, Yonsei University College of Medicine, Seoul, Republic of Korea; 3 Department of Rehabilitation, Hanshin University, Osan, Gyeonggi-do Republic of Korea; 4 Department of Pediatrics, Childhood Asthma Atopy Center, Humidifier Disinfectant Health Center, Asan Medical Center, University of Ulsan College of Medicine, Seoul, Republic of Korea; 5 Department of Pediatrics, Samsung Medical Center, Sungkyunkwan University School of Medicine, Seoul, Republic of Korea; 6 Environmental Health Center for Atopic Diseases, Samsung Medical Center, Seoul, Republic of Korea; 7 Department of Pediatrics, Yonsei University College of Medicine, Seoul, Republic of Korea; 8 Department of Pediatrics, CHA Gangnam Medical Center, CHA University School of Medicine, Seoul, Republic of Korea; 9 Department of Pediatrics, Seoul National University College of Medicine, Seoul, Republic of Korea

**Keywords:** Risk factors, Epidemiology

## Abstract

Air pollution may influence prenatal maternal stress, but research evidence is scarce. Using data from a prospective cohort study conducted on pregnant women (n = 2153), we explored the association between air pollution and perceived stress, which was assessed using the 14-item Perceived Stress Scale (PSS), among pregnant women. Average exposures to particulate matter with an aerodynamic diameter of < 2.5 µm (PM_2.5_) or < 10 µm (PM_10_), nitrogen dioxide (NO_2_), and ozone (O_3_) for each trimester and the entire pregnancy were estimated at maternal residential addresses using land-use regression models. Linear regression models were applied to estimate associations between PSS scores and exposures to each air pollutant. After adjustment for potential confounders, interquartile-range (IQR) increases in whole pregnancy exposures to PM_2.5_, PM_10_, and O_3_ in the third trimester were associated with 0.37 (95% confidence interval [CI] 0.01, 0.74), 0.54 (95% CI 0.11, 0.97), and 0.30 (95% CI 0.07, 0.54) point increases in prenatal PSS scores, respectively. Furthermore, these associations were more evident in women with child-bearing age and a lower level of education. Also, the association between PSS scores and PM_10_ was stronger in the spring. Our findings support the relationship between air pollution and prenatal maternal stress.

## Introduction

Maternal stress is relatively common during the prenatal period, with reported prevalences ranging from 12 to 92%^1–3^. Stress perceived by mothers during this period has been associated with adverse maternal (postpartum depression and suicide^4^) and fetal outcomes (preterm birth^5^, low birth weight^6^, congenital heart defects^7^, and impaired neonatal cognitive development^8^). In addition, higher risks of depression, impulsivity, and cognitive disorders have been reported in adolescence^9,10^ and schizophrenia in adulthood^11^ have been described in the offspring of women that experienced maternal stress during pregnancy. Therefore, identifying risk factors of prenatal maternal stress is a major public health goal.

Psychosocial stress can manifest in many forms, such as perceived stress, anxiety, or depression, and it has been reported that air pollution is associated with perceived stress^12^, mood^13^, suicide^14^, symptoms of depression^15^ and anxiety^16^, psychiatric emergency room visits^17^, and mental health disorders, as summarized in a meta-analysis^18^. Perceived stress is widely assessed using the Perceived Stress Scale (PSS; a measure of global stress), in which situations are appraised by individuals as stressful^19^. A longitudinal study of older men reported that exposure to air pollutants, including particulate matter with an aerodynamic diameter of < 2.5 µm (PM_2.5_) and nitrogen dioxide (NO_2_), was associated with higher PSS scores, particularly in cold months^12^. However, another study that explored the association between personal exposure to NO_2_ and PSS in healthy elderly reported non-significant results^13^.

Pregnant women are especially vulnerable to air pollution due to their increased ventilation rate for higher oxygen requirements of the developing fetus and decreased oxygen-binding capability^20^. In addition, the brains of pregnant women are more susceptible to environmental factors due to high metabolic demands^16^, as air pollutants may reach the brain by crossing the blood–brain barrier and trigger neuroinflammation, and subsequent pathological changes^21^. However, few studies have investigated the relationship between air pollution and maternal stress in pregnant women, and the studies undertaken have largely focused on postpartum depression or emotional stress^22–25^; other indicators of psychosocial stress, such as perceived stress, have not been studied in pregnant women. Nonetheless, it has been reported that the prevalence of maternal stress is higher in the prenatal than in the postnatal period^26,27^. Thus, we considered that delineating the nature of the relationship between air pollution and prenatal stress would increase understanding of the mechanism that links air pollution and affective disorders.

This study aimed to explore potential associations between exposure to air pollution during different trimesters and maternal PSS, which was assessed at the 36th week of pregnancy. We hypothesized that higher levels of air pollution would be associated with higher levels of perceived stress among pregnant women.

## Results

Table 1 presents a summary of the statistics of the study population (n = 2153). Mean subject age was approximately 33 years, and mean PSS score was 20.2 with a standard deviation of 7.2. 1314 (61.0%) were nulliparous, 2054 (95.4%) had educated to a higher level (college or above), and 32 (1.5%) were diagnosed with diabetes or hypertension in pregnancy. Most participants had high income [62.8% (≥ 4 million won/month)], and the majority were never smokers (92.3%).

**Table 1:** Characteristics of participants. *BMI* body mass index, *PSS* perceived stress scale, *SD* standard deviation. n = 2153.

Characteristics	Mean ± SD or n (%)
Age (years)	33.1 ± 3.6
**Age group**	
< 25	7 (0.3)
25–29	324 (15.1)
30–34	1121 (52.0)
35–39	604 (28.1)
≥ 40	97 (4.5)
**Pre-pregnancy BMI (kg/m**^**2**^**)**	
< 25	2012 (93.5)
≥ 25	141 (6.5)
**Parity**	
Nulliparous	1314 (61.0)
Parous	661 (30.7)
Missing	178 (8.3)
**History of smoking**	
Never	1988 (92.3)
Ever	165 (7.7)
**Drinking during pregnancy**	
No	1989 (92.4)
Yes	164 (7.6)
**Occupation**	
No	724 (33.6)
Yes	1429 (66.4)
**Education**	
Secondary school	99 (4.6)
College or university	1579 (73.3)
Graduate school	475 (22.1)
Gestational age (weeks)	39.3 ± 1.1
**Family income**	
High (≥ 4 million per month)	1353 (62.8)
Low (< 4 million per month)	800 (37.2)
**Asthma**	
No	2081 (96.7)
Yes	72 (3.3)
**Thyroid disease**	
No	2011 (93.4)
Yes	142 (6.6)
**Malignant tumor**	
No	2123 (98.6)
Yes	30 (1.4)
**Liver disease**	
No	2084 (96.8)
Yes	69 (3.2)
**Hypertension or diabetes mellitus**	
No	2121 (98.5)
Yes	32 (1.5)
**Season at delivery**	
Spring	465 (21.6)
Summer	432 (20.1)
Autumn	489 (22.7)
Winter	590 (27.4)
Missing	177 (8.2)
PSS score	20.2 ± 7.2

The distributions of air pollutant concentrations during the entire pregnancy and across the three trimesters are displayed in Table 2. The average concentrations of air pollutants during the first, second, and third trimesters were 27.64, 26.68, and 27.37 µg/m^3^, respectively, for PM_2.5_, 51.17, 49.40, and 50.70 µg/m^3^ for particulate matter with an aerodynamic diameter of < 10 µm (PM_10_), 35.04, 34.57, and 35.30 ppb for NO_2_, and 44.52, 43.45, and 41.49 ppb for ozone (O_3_). Correlations between pollutants by trimester are provided in Supplementary Table S1. Spearman’s correlation coefficients showed a strong relationship between PM_2.5_ and PM_10_ during pregnancy (Spearman’s correlation coefficient *r* = 0.82 to 0.91). PM_10_ showed a moderate positive correlation with NO_2_ (*r* = 0.30 to 0.54), whereas NO_2_ showed a moderate negative correlation with O_3_ (*r* =  − 0.46 to − 0.53). Furthermore, levels of air pollution in the second trimester were strongly correlated with the levels for the entire pregnancy (*r* = 0.80 to 0.88) (Supplementary Table S2).

**Table 2:** Distributions of maternal air pollution exposure levels for different pregnancy periods. *SD* standard deviation, *ppb* parts per billion.

Air pollutants	Mean ± SD	Min	25th	50th	75th	Max
**PM**_**2.5**_** (µg/m**^**3**^**)**						
1st trimester	27.64 ± 8.13	11.41	21.17	27.27	32.85	57.46
2nd trimester	26.68 ± 7.68	12.18	20.53	25.96	31.52	61.99
3rd trimester	27.37 ± 7.88	11.71	21.24	26.77	32.30	57.86
Whole pregnancy	27.19 ± 5.67	14.83	23.21	26.05	30.09	53.73
**PM**_**10**_** (µg/m**^**3**^**)**						
1st trimester	51.17 ± 12.40	24.25	40.95	51.77	61.23	88.82
2nd trimester	49.40 ± 11.59	25.73	39.31	49.09	58.58	81.48
3rd trimester	50.70 ± 12.04	24.48	40.78	51.19	59.91	85.67
Whole pregnancy	50.36 ± 6.47	34.60	45.80	50.17	54.66	73.93
**NO**_**2**_** (ppb)**						
1st trimester	35.04 ± 9.65	2.00	29.00	35.00	41.00	76.00
2nd trimester	34.57 ± 9.14	2.00	29.00	34.00	40.00	84.00
3rd trimester	35.30 ± 9.36	3.00	29.00	35.00	41.00	81.00
Whole pregnancy	34.94 ± 7.97	2.00	30.00	35.00	39.00	75.00
**O**_**3**_** (ppb)**						
1st trimester	44.52 ± 15.09	5.00	31.00	44.00	57.00	88.00
2nd trimester	43.45 ± 14.50	9.00	31.00	42.00	55.00	83.00
3rd trimester	41.49 ± 15.00	8.00	28.00	40.00	53.00	85.00
Whole pregnancy	43.22 ± 7.88	9.00	38.00	43.00	49.00	69.00

Associations between maternal air pollution exposure and PSS score are presented in Table 3. Analyses revealed significant associations between PSS scores and PM_10_ during the second trimester and entire pregnancy and between PSS scores and O_3_ during the third trimester before and after adjustment for relevant covariates. In the primary model, we observed significant positive associations between PSS scores and interquartile-range (IQR) increases in PM_2.5_ and PM_10_ in the first and second trimesters and entire pregnancy and between PSS scores and O_3_ in the third trimester. For example, an IQR increase in exposure to PM_2.5_ and PM_10_ in whole pregnancy was associated with 0.37 (95% confidence interval [CI] 0.01, 0.74) and 0.54 (95% CI 0.11, 0.97) point increases in PSS scores, respectively. Similarly, an IQR increase in exposure to O_3_ in the third trimester was associated with a 0.30 (95% CI 0.07, 0.54) point PSS score increase. No significant association was found between NO_2_ exposure and PSS scores. Regression coefficients were generally higher in the primary model (model 4), which was additionally adjusted for health behaviors and chronic disease variables, compared to the model that was adjusted for only demographic factors (model 2). When air pollutant exposures were categorized into quartiles, highest quartiles of maternal exposure to PM_10_ during the second trimester and O_3_ during the third trimester were found to be related to 1.07 (95% CI 0.10, 2.05) and 1.40 (0.48, 2.32) point increases in PSS scores, respectively (p for trend = 0.021 and 0.001, respectively) as compared with those in lowest quartiles (Supplementary Table S3). Subjects in the highest PM_2.5_ quartile for entire pregnancy had significantly higher PSS scores (p-value < 0.05), whereas those in the highest quartile for NO_2_ exposure during the first trimester showed only a marginally significant association (p-value < 0.1) (Supplementary Table S3).

**Table 3:** Associations between air pollution exposure (per IQR increase) and PSS scores among pregnant women in single-pollutant models. *CI* confidence interval, *IQR* interquartile range, *ppb* parts per billion, *PPS* perceived stress scale.

Air pollutants	Trimester	Model 1^a^	Model 2^b^	Model 3^c^	Model 4^d^
		β (95% CI)	β (95% CI)	β (95% CI)	β (95% CI)
PM_2.5_ (IQR: 6.88 µg/m^3^)	First	0.11 (− 0.15, 0.37)	0.13 (− 0.12, 0.39)	0.27 (− 0.01, 0.54)^+^	0.29 (0.01, 0.57)*
	Second	0.25 (− 0.02, 0.53)^+^	0.29 (0.02, 0.56)*	0.32 (0.03, 0.61)*	0.36 (0.06, 0.65)*
	Third	0.04 (− 0.23, 0.30)	0.04 (− 0.22, 0.31)	− 0.04 (− 0.31, 0.24)	− 0.03 (− 0.31, 0.24)
	Pregnancy	0.28 (− 0.09, 0.64)	0.32 (− 0.05, 0.69)^+^	0.32 (− 0.05, 0.68)^+^	0.37 (0.01, 0.74)*
PM_10_ (IQR: 8.86 µg/m^3^)	First	0.12 (− 0.10, 0.34)	0.14 (− 0.08, 0.35)	0.26 (0.02, 0.51)*	0.29 (0.05, 0.53)*
	Second	0.25 (0.01, 0.48)*	0.28 (0.05, 0.51)*	0.31 (0.04, 0.59)*	0.36 (0.08, 0.63)*
	Third	0.01 (− 0.21, 0.24)	0.01 (− 0.21, 0.24)	− 0.11 (− 0.35, 0.13)	− 0.11 (− 0.35, 0.14)
	Pregnancy	0.47 (0.05, 0.88)*	0.53 (0.11, 0.94)*	0.47 (0.04, 0.90)*	0.54 (0.11, 0.97)*
NO_2_ (IQR: 9.0 ppb)	First	− 0.01 (− 0.30, 0.27)	0.06 (− 0.22, 0.35)	0.17 (− 0.11, 0.45)	0.18 (− 0.10, 0.46)
	Second	0.02 (− 0.28, 0.32)	0.12 (− 0.18, 0.42)	0.13 (− 0.18, 0.45)	0.13 (− 0.19, 0.45)
	Third	− 0.24 (− 0.53, 0.05)	− 0.17 (− 0.46, 0.12)	− 0.15 (− 0.43, 0.14)	− 0.16 (− 0.45, 0.12)
	Pregnancy	− 0.10 (− 0.44, 0.25)	0.01 (− 0.33, 0.36)	0.07 (− 0.27, 0.41)	0.07 (− 0.27, 0.41)
O_3_ (IQR: 11.0 ppb)	First	− 0.10 (− 0.33, 0.12)	− 0.13 (− 0.35, 0.09)	− 0.13 (− 0.36, 0.09)	− 0.15 (− 0.38, 0.08)
	Second	0.04 (− 0.19, 0.27)	− 0.01 (− 0.22, 0.24)	0.18 (− 0.11, 0.47)	0.19 (− 0.10, 0.48)
	Third	0.30 (0.07, 0.52)**	0.30 (0.08, 0.52)**	0.30 (0.06, 0.54)*	0.30 (0.07, 0.54)*
	Pregnancy	0.25 (− 0.18, 0.67)	0.18 (− 0.24, 0.61)	0.32 (− 0.14, 0.78)	0.32 (− 0.14, 0.77)

^+^p-value < 0.1. *p-value < 0.05. **p-value < 0.01.

^a^Model 1: unadjusted.

^b^Model 2: Model 1 + maternal age, education, occupation, and income.

^c^Model 3: Model 2 + gestational age, maternal smoking, drinking during pregnancy, parity, pre-pregnancy BMI, and season at delivery.

^d^Model 4: Model 3 + asthma, thyroid disease, malignant tumors, liver disease, and hypertension or diabetes.

**Figure 1:**
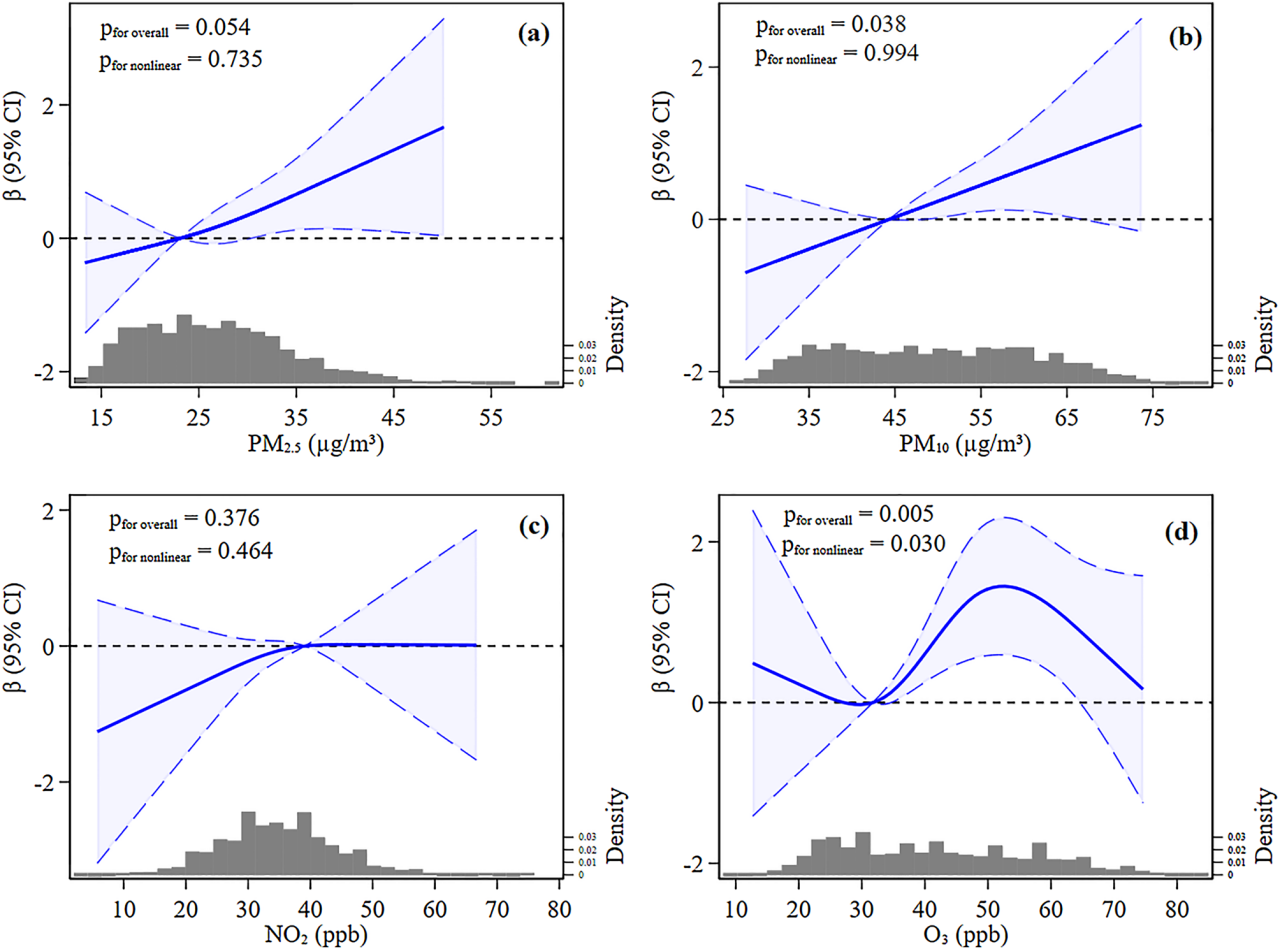
Nonlinear effects of PM_2.5_ (**a**), PM_10_ (**b**), NO_2_, (**c**) and O_3_ (**d**) on PSS scores. Point estimates (solid line) and 95% confidence intervals (long dashed lines) were obtained by restricted cubic splines with three knots at the 10th, 50th, and 90th percentiles of PM_2.5_, PM_10_, and NO_2_ distributions and four knots at the 5th, 35th, 65th, and 95th percentiles of the O_3_ distribution. PM_2.5_ and PM_10_ during the second trimester, NO_2_ during the first trimester, and O_3_ during the third trimester were used for spline analyses. Reference PM_2.5_, PM_10_, NO_2_, and O_3_ values for these plots with β fixed at 0.0 were 22.4 μg/m^3^, 43.9 μg/m^3^, 39.0 parts per billion (ppb), and 31.0 ppb, respectively. Models adjusted for maternal age, education, occupation, gestational age, maternal smoking, drinking during pregnancy, parity, pre-pregnancy BMI, season at delivery, income, asthma, thyroid disease, malignant tumors, liver disease, and hypertension or diabetes. Histograms show the distributions of PM_2.5_ (**a**), PM_10_ (**b**), NO_2_ (**c**), and O_3_ (**d**) exposures.

Spline analyses showed that increases in mean PM_10_ concentration during the second trimester and O_3_ concentration during the third trimester were associated with higher PSS scores (p for overall association = 0.038 and 0.005, respectively) (Fig. 1b,d): PM_2.5_ exposures during the second trimester were only marginally associated with PSS scores (p for overall association = 0.054) (Fig. 1a). Spline analyses showed linear dose–response relationships between PM_2.5_ and PM_10_ concentrations during the second trimester and PSS scores (p for nonlinear = 0.735 and 0.994, respectively). In contrast, the association between maternal O_3_ exposure during the third trimester and PSS scores was nonlinear (p for nonlinear = 0.030). The overall association between NO_2_ exposure during the first trimester and PSS scores was insignificant (p for overall = 0.376) and did not significantly deviate from linearity (p for nonlinear = 0.464) (Fig. 1c), which was consistent with estimates obtained using the other models (Supplementary Fig. S1).

**Figure 2:**
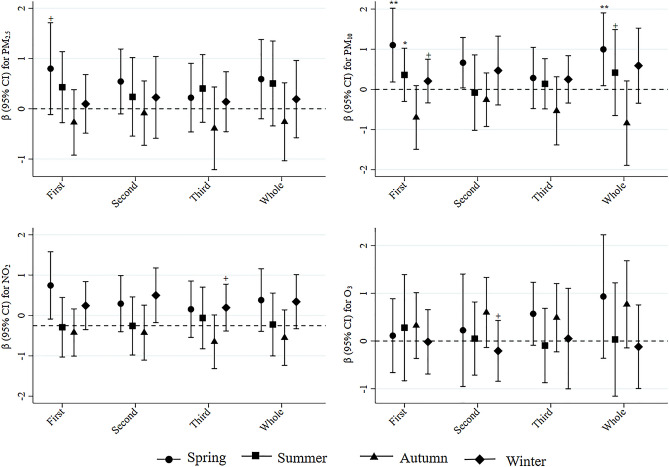
Adjusted differences in PSS scores per IQR increase in air pollutants stratified by season. Analyses were adjusted for maternal age, education, smoking, occupation, drinking during pregnancy, parity, pre-pregnancy BMI, gestational age, income, asthma, thyroid disease, malignant tumors, liver diseases, and hypertension or diabetes. ^+^p for interaction < 0.1. *p for interaction < 0.05. **p for interaction < 0.01.

Figure 2 shows evidence of effect modification of associations between air pollution and PSS scores by season. Significant interactions were observed between spring and PM_10_ exposures during the first trimester and entire pregnancy (p for interactions < 0.01); positive associations between PSS scores and PM_10_ were significant in spring, but not in other seasons. Analyses of effect modification by maternal age, parity, education, and income are reported in Supplementary Fig. S2. In age-stratified models, significant associations were observed between PM_10_ exposures during the first and second trimesters and entire pregnancy and PSS scores and between O_3_ exposures during the third trimester and PSS scores in pregnant women aged < 35 years, but not in women ≥ 35 years old. A significant interaction was observed between O_3_ exposure during the third trimester and age (p for interaction < 0.05), while interactions between PM_10_ exposure in the second trimester and entire pregnancy and age were marginally significant (p for interactions < 0.1). Significant positive associations were observed between PSS scores and PM_2.5_ exposures during the second trimester and entire pregnancy only in women with less than graduate education (p for interactions < 0.05). For parity and income, the associations of PM_2.5_, PM_10_, and O_3_ with PSS were greater for nulliparous women and women with low incomes, but p-values for interaction terms were not significant (p > 0.05).

Sensitivity analyses revealed relationships between maternal exposure to PM_10_ during the second trimester and entire pregnancy and PSS scores and between maternal exposure to O_3_ during the third trimester and PSS scores remained significant after adding other pollutants to the primary model (Table 4). A separate analysis was performed using the primary model after excluding mothers with a chronic health condition but this did not materially change estimates of relationships between maternal exposure to air pollution and PSS scores (Supplementary Table S4). Furthermore, similar findings were obtained when these relationships were examined using the multiple imputation technique (Supplementary Table S5). The E-values with lower 95% CI and relative risks, as determined using the primary model, are provided in Supplementary Table S6. These results indicate that our conclusions are robust to unmeasured confounding bias.

**Table 4:** Multi-pollutant model of associations between exposure to air pollutants (per IQR increase) and PSS scores among pregnant women.

Trimester	+ PM_10_	+ NO_2_	+ O_3_	+ PM_10_ + NO_2_ + O_3_
	β (95% CI)^a^	β (95% CI)^a^	β (95% CI)^a^	β (95% CI)^a^
**First**				
PM_10_	–	0.29 (0.001, 0.58)*	0.28 (− 0.001, 0.56)^+^	0.28 (− 0.02, 0.59)^+^
NO_2_	− 0.01 (− 0.34, 0.33)	–	0.11 (− 0.23, 0.45)	− 0.02 (− 0.38, 0.34)
O_3_	− 0.02 (− 0.28,0.24)	− 0.10 (− 0.37, 0.18)	–	− 0.03 (− 0.31, 0.26)
**Second**				
PM_10_	–	0.36 (0.07, 0.65)*	0.34 (0.06, 0.61)*	0.31 (0.01, 0.62)*
NO_2_	− 0.01 (− 0.35, 0.33)	–	0.23 (− 0.11, 0.57)	0.07 (− 0.31, 0.45)
O_3_	0.13 (− 0.16, 0.43)	0.26 (− 0.05, 0.58)^+^	–	0.16 (− 0.17, 0.49)
**Third**				
PM_10_	–	− 0.05 (− 0.33, 0.23)	0.06 (− 0.22, 0.33)	0.05 (− 0.25, 0.34)
NO_2_	− 0.14 (− 0.47, 0.19)	–	0.06 (− 0.28, 0.41)	0.04 (− 0.32, 0.41)
O_3_	0.33 (0.06, 0.60)*	0.33 (0.04, 0.62)*	–	0.35 (0.04, 0.65)*
**Pregnancy**				
PM_10_	–	0.56 (0.11, 1.01)*	0.54 (0.11, 0.97)*	0.52 (0.07, 0.98)*
NO_2_	− 0.07 (− 0.42, 0.29)	–	0.21 (− 0.17, 0.59)	0.06 (− 0.34, 0.46)
O_3_	0.33 (− 0.13, 0.78)	0.44 (− 0.07, 0.95)^+^	–	0.36 (− 0.15, 0.88)

^+^p-value < 0.1. *p-value < 0.05.

^a^Multi-pollutant model was further adjusted for the effects of other air pollutants in the same time window using the adjusted single-pollutant model 4 shown in Table 3.

## Discussion

In this prospective cohort study of pregnant women, we investigated the association between exposure to air pollution and maternal PSS scores. We found that maternal exposure to PM_2.5_ or PM_10_ during the first and second trimesters and entire pregnancy and exposure to O_3_ during the third trimester were significantly associated with higher perceived stress during the third trimester, as determined using single-pollutant models, and that associations between PM_10_ exposure during the second trimester and entire pregnancy and O_3_ during the third trimester were also significantly associated with PSS scores using multi-pollutant models. In addition, we found evidence of effect modifications of air pollution-perceived stress associations by season, maternal age, and education.

Experimental research provides plausible results for potential associations between air pollution and stress^28^. Yokota et al. showed that exposure to diesel exhaust particles during pregnancy decreased serotonin in mice, which can lead to depressive behaviors^29^. Furthermore, epidemiological studies have reported an association between air pollution and specific perceived stress, such as perceived air quality among pedestrians^30^ and non-specific perceived stress in elderly men^12^. In the present study, we sought to detect changes in non-specific perceived stress in pregnant women. This type of stress indicates that individual perceptions of air quality are unlikely to change due to the range of air pollution levels. In addition, perceived stress is considered an intermediate stage in the association between exposure to air pollution and affective psychiatric disorders. Pregnant women are particularly susceptible to the adverse effects of air pollution. Four studies have so far investigated the relationship between exposure to air pollution during pregnancy and maternal mental health during prenatal and postnatal periods^22–25^. One of these studies reported a link between air pollution and maternal depression during pregnancy^22^, two supported a link between exposure to PM_2.5_ in pregnancy and postpartum depression^23,24^, and other concluded that exposure to PM_2.5_ or NO_2_ from mid to late pregnancy is associated with higher maternal Global Severity-Indices, which indicated higher levels of emotional stress during pregnancy^25^. However, no study has examined the association between exposure to air pollution in different trimesters of pregnancy and prenatal maternal stress.

Stratified analyses showed PM_10_ exposure had a significant positive effect on PSS scores during spring, but not during other seasons. This finding is consistent with that of a previous Korean study, which reported a stronger association between PM_10_ exposure and suicide during spring^14^. The average PM_10_ concentration is relatively high in Seoul during the spring^31^, and thus, pregnant women are probably more likely to perceive air pollution in the spring. However, some studies have reported stronger associations between particulate air pollution and maternal perceived or emotional stress in the cold season^12,25^, which is not in line with our results. This inconsistency is possibly due to seasonal and geographical variations of air pollutant levels and different weather conditions and lifestyles^32^.

Interestingly, we observed a stronger positive association between PSS scores and PM_2.5_ and PM_10_ exposures in pregnant women of lower socio-economic status, particularly in women with a lower education level. In Korea, air pollution is known to be spatially associated with lower socio-economic status, which includes considerations of income and educational attainment^33,34^. In addition, individuals with low incomes and less education are more likely to have higher perceived stress levels^35^ and to be more susceptible to the effects of air pollution. Therefore, the combination of more pollution exposure and the influence of indicators of lower socio-economic status may explain the strong associations observed between particulate air pollution and PSS scores among pregnant women in the present study. In addition, women aged < 35 years showed a significant association between air pollution exposure and perceived stress, whereas older women (≥ 35 years) did not. Young pregnant women are more likely to develop stress-related disorders^36^, which may contribute to the increased vulnerability to air pollution.

Although the mechanisms whereby air pollution affects maternal stress during pregnancy are unclear, we believe our results are biologically plausible. Research has demonstrated that exposure to particulate matter increases inflammation in mouse brains^37^, and chronic brain inflammation is associated with reactive oxygen species formation and oxidative stress, which have been suggested to be biological pathways that lead to mental disorders^38,39^. Higher energy requirements and physiological changes render pregnant women more vulnerable to oxidative stress than the general population^40^. In addition, air pollution has been associated with increased inflammatory markers and altered cytokine production^41^, and maternal exposure to air pollution has also been suggested to cause stress due to activation of the hypothalamic–pituitary–adrenal axis^42^, which is known to be associated with stress and stress-related disorders^43,44^. These findings indicate a possible mediator for the neurotoxic effects of air pollutants on maternal stress.

The major strengths of our study were the use of validated models for assessing the effects of exposure to air pollution and the adjustment for multiple confounders. In addition, the study shows individuals’ characteristics have modifying effects on the association between air pollution and PSS scores, which provide insight of susceptibility. Furthermore, multiple sensitivity analysis demonstrated the robustness of our findings, and the use of multi-pollutant models enabled us to conclude that PM_10_ and O_3_ exposure are individually associated with higher perceived stress independently of each other. To the best of our knowledge, the present study is the first to investigate the relation between air pollution during pregnancy and the subsequent development of prenatal maternal stress. However, this study has several limitations. First, air pollution estimates were based on maternal residential addresses, and geospatial data on maternal residential history, movement patterns, and time spent indoors and outdoors were unavailable, which may have introduced bias into our model of air pollution exposure, particularly if women with perceived stress have different local mobility patterns. Previous research has indicated maternal mobility during pregnancy is usually limited and generally restricted to residential areas^45^, whereas air pollution tends to be homogenous across local areas. Thus, we believe that movement patterns would have little effect on our exposure estimates. Second, although we controlled for several important potential confounders, our results may have been biased by residual confounding due to unmeasured confounders. For example, it is possible that adjustment for physical activity might have attenuated our results to the null, as inactivity would have reduced exposure to air pollution and been associated with higher levels of perceived stress. Adjustment for socioeconomic status, maternal occupation, and pre-pregnancy BMI may have limited some of the potential confounding caused by physical activity, and therefore, reduced the level of residual confounding. In addition, other factors such as residential noise, meteorologic conditions, and exposure to indoor pollutants might have contributed to confounding or introduced measurement errors that might explain our finding of a positive association between air pollution and PSS scores. To address this issue we evaluated E-values to assess the strength of the relationship and to determine whether a hypothetical unmeasured confounder might have markedly altered our results, but the values obtained suggested they were unlikely to have had a major impact. Third, perceived stress was assessed using self-reports only once during pregnancy, and thus, the responses obtained would not have represented maternal stress status throughout pregnancy. Finally, while the homogenous makeup of the Cohort for Childhood Origin of Asthma and allergic diseases (COCOA) study in the Korean population provided excellent internal validity, its generalizability in other populations may be limited.

Nonetheless, the findings of this study have substantial public health implications because perceived stress is a risk factor of affective disorders^4^, cardiovascular disease, and mortality^46–48^. Previous studies have reported that the risk of physical health problems associated with air pollution may be increased by perceived stress^49,50^. However, our findings suggest perceived stress may also be a mediator, which should be taken into consideration. Our findings indicate that air pollution might be a modifiable risk factor of perceived stress during pregnancy. Interventions to improve ambient air quality may reduce women’s vulnerability to elevated stress during pregnancy and diminish the risk of poor physical health outcomes associated with stress. In addition, our results highlight the need for perceived stress assessment during pregnancy in areas of high air pollution to prevent exacerbations of affective symptoms during the perinatal period.

## Conclusions

Our findings suggest maternal exposures to PM_2.5_, PM_10_, and O_3_ are related to higher levels of perceived stress among pregnant women. Pregnant women aged less than 35 years and a lower level of education may be more susceptible to air pollution. In addition, we found that the association between PM_10_ exposure and PSS scores was stronger in the spring. Future studies are warranted to determine how air pollution exacerbates perceived stress and to assess the effects of perceived stress on associations between air pollution and affective disorders.

## Methods

### Study participants

The COCOA was a prospective cohort study of mothers and children conducted at five medical centers and eight public health centers in the Seoul metropolitan area (2007–2015)^51^. A total of 3102 pregnant women of gestational age less than 26 weeks were recruited for the COCOA study. For this analysis, women without PSS data (n = 638) were excluded. Women with multiple records (n = 86) and missing information on residential address (n = 76) were not included in the study. To avoid potential confounding, mothers of preterm babies (n = 75) were excluded. Those with missing covariate information (n = 74) were also excluded. Overall, 949 women were excluded, and the final sample consisted of 2153 pregnant women. Exclusion criteria details are presented in Supplementary Fig. S3. With the exception of family income, no significant differences were observed those included or excluded (Supplementary Table S7). Data were collected in accordance with relevant guidelines and regulations, and all participants provided written informed consent before being included in the investigation. Ethical approval was provided by the Asan Medical Center (IRB No. 2008-0616), Samsung Medical Center (IRB No. 2009-02-021), Yonsei University (IRB No. 4-2008-0588), the CHA Medical Center (IRB No. 2010-010), and Seoul National University (IRB No. 1401-086-550).

### Assessment of prenatal maternal stress

Maternal stress was assessed during the third trimester of pregnancy (36th week of pregnancy) using PSS, which is a 14-item questionnaire intended to assess perception of stress based on responses to seven positively and seven negatively stated questions^19,52^. Responses were rated using a Likert five-point scale, which ranged from “never” (0) to “very often” (4). The scores for the seven positively stated items in the 14-items scale were negated, and total scores were obtained by summing all items. Thus, possible total scores ranged from 0 to 56, and higher scores indicated higher levels of perceived stress. The PSS has been shown to be a valid instrument for the assessment of mental stress among pregnant women (Cronbach’s alpha = 0.88)^53^. In the current study, PSS scores were normally distributed and exhibited excellent reliability (Cronbach’s alpha = 0.884).

### Air pollution exposure

The land use regression (LUR) model was used to predict maternal exposures to PM_2.5_, PM_10_, NO_2_, and O_3_. Air pollution data were obtained from the Korean Ministry of the Environment (http://www.airkorea.or.kr/web), which monitored 24-h average concentrations of PM_2.5_, PM_10_, NO_2_, and O_3_ in air daily at a maximum of 40 regulatory sites in Seoul from 2007 to 2015. Monthly exposures to particulate matter (PM_2.5_ and PM_10_), NO_2_, and O_3_ at maternal residential addresses were estimated using the LUR model, as previously described^54,55^. This model uses several geographical variables, which include traffic indicators, surrounding‐land use, topography, and spatial trends, and the final LUR model included lengths of roads, traffic intensities on nearest roads, total heavy‐duty traffic on all roads, and a variable that represents spatial trends. Model performance was assessed using leave-one-out cross-validation (LOOCV). The model explained 66–81% of the variability in measured PM_2.5_, PM_10_, NO_2_, and O_3_ levels, and the predicted values agreed well with measured values, as we previously reported^56,57^. Model-adjusted R^2^ and LOOCV R^2^ values for PM_2.5_, PM_10_, NO_2_, and O_3_ were 0.66 and 0.56, 0.69 and 0.60, 0.79 and 0.73, and 0.81 and 0.77, respectively.

To investigate potential critical exposures during pregnancy, we calculated the average concentrations of PM_2.5_, PM_10_, NO_2_, and O_3_ for the three trimesters (1st trimester from 1 to 13 weeks, 2nd trimester from 14 to 27 weeks, and 3rd trimester from 28 weeks to until birth) and entire pregnancy.

### Covariates

Potential confounding variables, including maternal age at delivery, maternal education, occupation during pregnancy (yes vs. no), parity (nulliparous vs. parous), pre-pregnancy body mass index (BMI) (kg/m^2^: ≥ 25 vs. < 25), smoking history (ever vs. never), family income, and drinking during pregnancy (yes vs. no), were ascertained at baseline. Maternal age was divided into five categories (< 25, 25–29, 30–34, 35–39, and ≥ 40), and mother’s education was categorized as secondary school, college or university, and graduate school. Family income was dichotomized as high (≥ 4 million Korean won per month) or low (< 4 million Korean won per month)^58^. The data on gestational age in weeks was obtained from medical records at delivery. We also obtained medical histories according to physicians’ diagnoses, including hypertension or diabetes mellitus, asthma, thyroid diseases, malignant tumors, and liver diseases. We used seasons of child birth to investigate seasonal variations in perceived stress during the third trimester. The seasons at delivery were defined as; spring (March to May), summer (June to August), autumn (September to November), and winter (December to February) based on the climate characteristics in South Korea. Previous studies indicate that these variables are related to psychosocial stress and may affect the estimates of associations between exposure to air pollution and perceived stress^1,12,59^.

### Statistical analysis

The statistical analysis was performed using STATA version 16.0 (Stata Corporation). Descriptive statistics are presented as mean ± standard deviations (SDs) or frequencies (%). Spearman’s correlation was used to examine the correlations among air pollutants. Linear regression models were used to assess associations between PSS scores and air pollutant levels averaged over each trimester or whole pregnancy and PSS scores. The regression coefficients were estimated by IQR increases in pollutant concentrations averaged over the pregnancy. Model 1 was used to investigate bivariate associations between PSS scores and air pollutant levels measured during pregnancy. Model 2 was adjusted for age, education, occupation, and income, whereas Model 3 was additionally adjusted for lifestyle and maternal characteristics, which included gestational age, smoking, drinking during pregnancy, parity, pre-pregnancy BMI, and season at delivery. In Model 4, the primary model, we added adjustment for chronic health conditions, which included asthma, thyroid disease, malignant tumors, liver disease, and hypertension or diabetes. In addition to modeling air pollution as continuous variables, it was also modeled as categorical variables by quartiles of air pollution exposure using linear regression for the primary model. We tested for linear trends across quartiles by assigning median air pollutant values to quartile as continuous variables in separate regression models. The results from quartile analysis are reported in Supplementary Table S3.

To explore the potential nonlinear associations between maternal air pollution exposure and PSS scores, we performed restricted cubic spline analysis based on linear regression model^60^. The optimal number of knots for analysis was determined using the Akaike information criterion (AIC). Nonlinearity was assessed by testing the regression coefficients of nonlinear terms, and a p-value of nonlinearity of < 0.05 was considered to indicate a nonlinear association: the overall association indicated that the regression coefficients of both linear and non-linear terms of the factor were equal to zero.

In addition, stratified analysis was conducted to examine the effect modification by maternal age, parity, education, income, and season at delivery on the relation between air pollution and perceived stress. To evaluate the significance of effect modification on the multiplicative scale, we included an interaction (product) term between air pollution exposure and each of these five characteristics in our primary model.

Several sensitivity analyses were conducted. First, to test for stability of the effects after controlling for other pollutants, we constructed multi-pollutant models for pregnancy exposure for PM_10_, NO_2_, and O_3_ but not for PM_2.5_ due to its high correlation with PM_10_ (Supplementary Table S1). Second, we reanalyzed the primary model after excluding women with a chronic health condition. Third, we reanalyzed the primary model using multiple imputation technique. Finally, we evaluated the robustness of our results with respect to unmeasured cofounding by calculating the E-values^61^, which indicate the minimum strength of an association that unmeasured confounders must have to explain away associations between exposure (air pollution) and outcome (PSS scores). The E-value of the relative risk estimate for air pollution was calculated using the primary model.

## Supplementary Information

Supplementary Information.

## Data Availability

The datasets generated and/or analyzed during the current study are not publicly available, but are available from the corresponding author on reasonable request.
