# Supplementary material for: Association between ambient air pollution and perceived stress in pregnant women
Source: Sci Rep. 2021 Dec 6;11:23496. doi: 10.1038/s41598-021-02845-4 (PMC8648786; doi:10.1038/s41598-021-02845-4)
Supplement: Supplementary file 1 — Supplementary Information. [file 41598_2021_2845_MOESM1_ESM.pdf]

Supplementary Information

For

Association between ambient air pollution and perceived stress in pregnant women

Dirga Kumar Lamichhane[1], Dal-Young Jung[1], Yee-Jin Shin[2], Kyung-Sook Lee[3], So-Yeon Lee[4], Kangmo Ahn[5,6], Kyung Won Kim[7], Youn Ho Shin[8], Dong In Suh[9], Soo-Jong Hong[4] & Hwan-Cheol Kim[1]

[1]Department of Occupational and Environmental Medicine, Inha University School of Medicine, Incheon, Republic of Korea

[2]Department of Psychiatry, Yonsei University College of Medicine, Seoul, Republic of Korea

[3]Department of Rehabilitation, Hanshin University, Gyeonggi-do, Republic of Korea

[4]Department of Pediatrics, Childhood Asthma Atopy Center, Humidifier Disinfectant Health Center, Asan Medical Center, University of Ulsan College of Medicine, Seoul, Republic of Korea

[5]Department of Pediatrics, Samsung Medical Center, Sungkyunkwan University School of Medicine, Seoul, Republic of Korea

[6]Environmental Health Center for Atopic Diseases, Samsung Medical Center, Seoul, Republic of Korea

[7]Department of Pediatrics, Yonsei University College of Medicine, Seoul, Republic of Korea

[8]Department of Pediatrics, CHA Gangnam Medical Center, CHA University School of Medicine, Seoul, Republic of Korea

[9]Department of Pediatrics, Seoul National University College of Medicine, Seoul, Republic of Korea

Table S1. Spearman correlations among maternal air pollutants by trimesters and entire pregnancy

	$PM_{2.5}$	PM_{10}	NO_2	O_3
1st trimester				
$PM_{2.5}$	1.00			
PM_{10}	0.91*	1.00		
NO_2	0.49*	0.54*	1.00	
O_3	−0.24*	−0.24*	−0.50*	1.00
2nd trimester				
$PM_{2.5}$	1.00			
PM_{10}	0.91*	1.00		
NO_2	0.44*	0.49*	1.00	
O_3	−0.26*	−0.27*	−0.52*	1.00
3rd trimester				
$PM_{2.5}$	1.00			
PM_{10}	0.90*	1.00		
NO_2	0.44*	0.47*	1.00	
O_3	−0.24*	−0.23*	−0.53*	1.00
Whole pregnancy				
$PM_{2.5}$	1.00			
PM_{10}	0.82*	1.00		
NO_2	0.28*	0.30*	1.00	
O_3	−0.20*	−0.13*	−0.46*	1.00

*p-value < 0.05.

Table S2. Spearman correlations of each air pollutant with itself across three trimesters and entire pregnancy

	1st trimester	2nd trimester	3rd trimester	Whole pregnancy
$PM_{2.5}$				
1st trimester	1.00			
2nd trimester	0.30*	1.00		
3rd trimester	−0.13*	0.32*	1.00	
Whole pregnancy	0.60*	0.83*	0.52*	100
PM_{10}				
1st trimester	1.00			
2nd trimester	0.08*	1.00		
3rd trimester	−0.49*	0.12*	1.00	
Whole pregnancy	0.41*	0.80*	0.31*	1.00
NO_2				
1st trimester	1.00			
2nd trimester	0.56*	1.00		
3rd trimester	0.31*	0.57*	1.00	
Whole pregnancy	0.77*	0.88*	0.75*	1.00
O_3				
1st trimester	1.00			
2nd trimester	0.12*	1.00		
3rd trimester	−0.61*	0.11*	1.00	
Whole pregnancy	0.35*	0.85*	0.25*	1.00

*p-value < 0.05.

Table S3. Associations between quartiles of maternal air pollution exposure and PSS scores

Air pollutants	1st trimester	2nd trimester	3rd trimester	Whole pregnancy
$PM_{2.5}$				
1st quartile	Reference	Reference	Reference	Reference
2nd quartile	−0.28 (−1.14, 0.57)	0.09 (−0.76, 0.94)	−0.66 (−1.50, 0.19)	0.73 (−0.11, 1.57)
3rd quartile	0.79 (−0.10, 1.68) +	0.40 (−0.49, 1.30)	−0.29 (−1.16, 0.58)	0.36 (−0.49, 1.21)
4th quartile	0.53 (−0.39, 1.46)	0.77 (−0.15, 1.69) +	−0.42 (−1.31, 0.46)	0.91 (0.06, 1.75) *
p for trend	0.090	0.076	0.104	0.081
PM_{10}				
1st quartile	Reference	Reference	Reference	Reference
2nd quartile	−0.07 (−0.93, 0.79)	0.47 (−0.38, 1.33)	−0.69 (−1.54, 0.15)	0.22 (−0.62, 1.06)
3rd quartile	0.50 (−0.41, 1.41)	0.92 (0.03, 1.81) *	−0.79 (−1.67, 0.09) +	0.93 (0.08, 1.78) *
4th quartile	0.69 (−0.26, 1.63)	1.07 (0.10, 2.05) *	−0.41 (−1.33, 0.51)	0.72 (−0.17, 1.60)
p for trend	0.088	0.021	0.358	0.051
NO_2				
1st quartile	Reference	Reference	Reference	Reference
2nd quartile	0.42 (−0.39, 1.23)	0.04 (−0.79, 0.87)	−0.32 (−1.16, 0.52)	−0.22 (−1.01, 0.58)
3rd quartile	0.69 (−0.14, 1.53)	0.52 (−0.33, 1.37)	−0.61 (−1.42, 0.20)	0.36 (−0.54, 1.26)
4th quartile	0.75 (−0.10, 1.59) +	0.15 (−0.76, 1.06)	−0.52 (−1.36, 0.31)	0.38 (−0.46, 1.23)
p for trend	0.060	0.596	0.162	0.234
O_3				
1st quartile	Reference	Reference	Reference	Reference
2nd quartile	−0.14 (−0.98, 0.70)	0.13 (−0.74, 1.00)	0.36 (−0.48, 1.21)	0.86 (0.05, 1.68) *
3rd quartile	−0.58 (−1.42, 0.25)	−0.19 (−1.19, 0.80)	0.92 (0.05, 1.78) *	0.23 (−0.64, 1.11)
4th quartile	−0.36 (−1.25, 0.53)	0.71 (−0.34, 1.76)	1.40 (0.48, 2.32) **	1.00 (0.06, 1.94) *
p for trend	0.272	0.209	0.001	0.093

Quartiles of maternal air pollution exposure levels were categorized based on trimester-specific cut points in Table 2 (25th, 50th, and 75th percentiles in each trimester and entire pregnancy). p-values for trend were derived by assigning the median value of each quartile as a continuous variable. Models adjusted for maternal age, education, occupation, gestational age, maternal smoking, drinking during pregnancy, pre-pregnancy BMI, parity, season at delivery, income, asthma, thyroid disease, malignant tumors, liver disease, and hypertension or diabetes.

+ p-value < 0.1. * p-value < 0.05. ** p-value < 0.01.

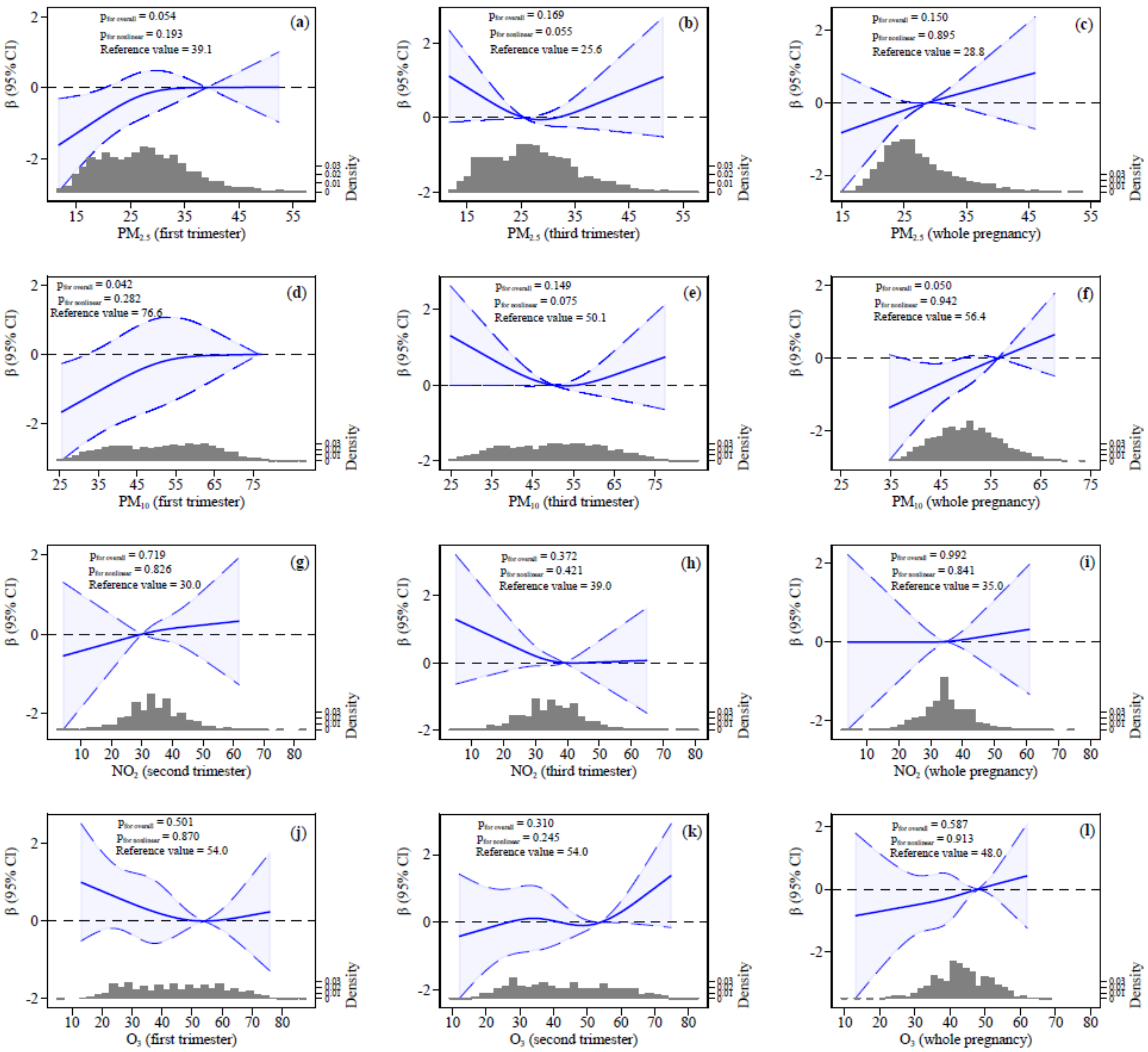

Figure S1. Nonlinear effects of $PM_{2.5}$ (a, b and c), PM_{10} (d, e and f), NO_2 (g, h, and i) and O_3 (j, k, and l) on the PSS score. Point estimates (solid line) and 95% confidence intervals (long dashed lines) were obtained by restricted cubic splines with three knots at the 10th, 50th, and 90th percentiles of $PM_{2.5}$, PM_{10}, and NO_2 distributions and four knots at the 5th, 35th, 65th, and 95th percentiles of the O_3 distribution. Models adjusted for maternal age, education, occupation, gestational age, maternal smoking, drinking during pregnancy, parity, pre-pregnancy BMI, season at delivery, income, asthma, thyroid disease, malignant tumors, liver disease, and hypertension or diabetes. Histograms show the distributions of $PM_{2.5}$ (a, b, and c), PM_{10} (d, e, and f), NO_2 (g, h, and i), and O_3 (j, k, and l) exposures.

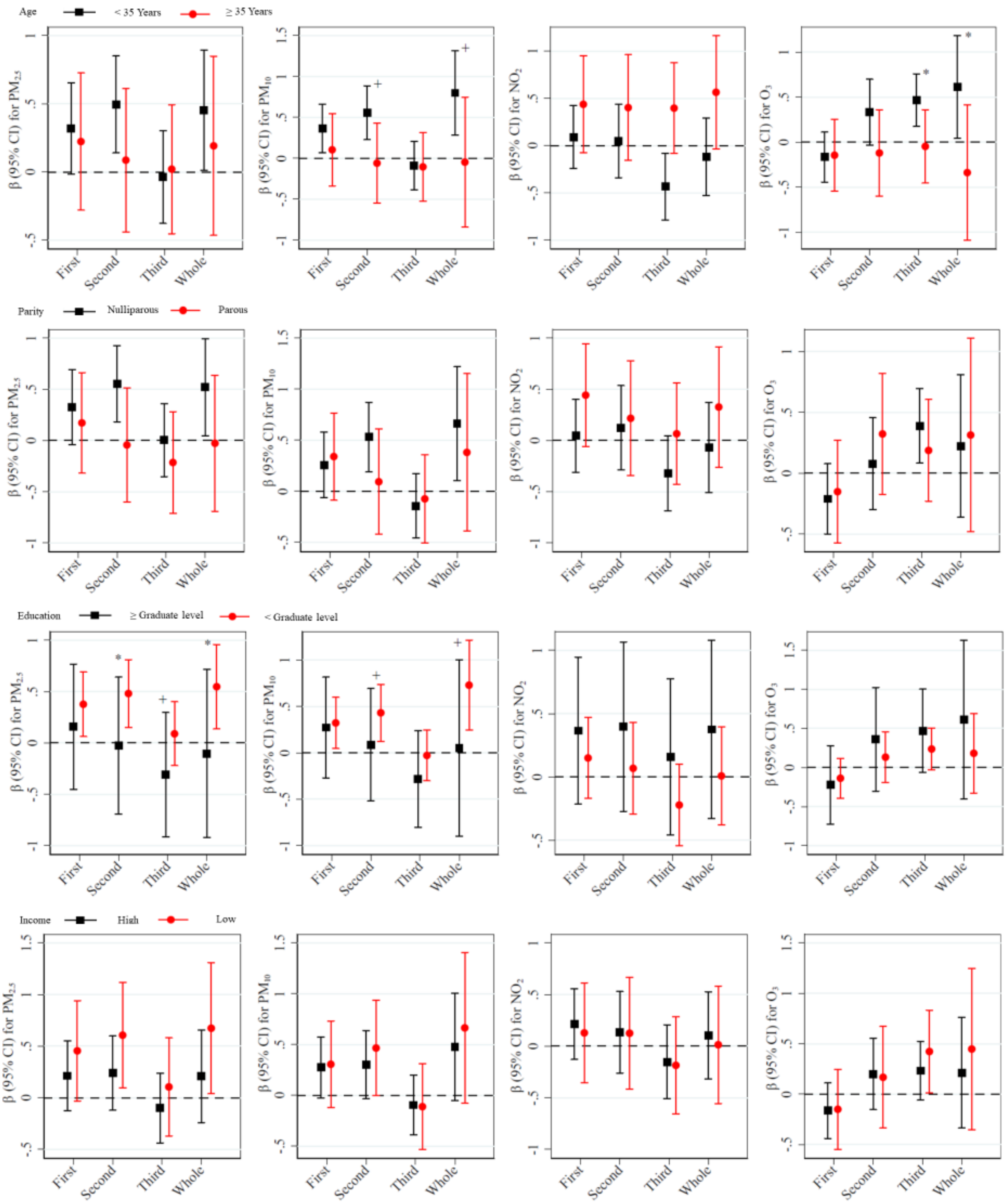

Figure S2. Stratified analysis of air pollution and PSS scores. Adjusted difference in PSS scores per IQR increase in air pollutants stratified by age, parity, education, and income. Models adjusted for maternal age, education, occupation, gestational age, maternal smoking, drinking during pregnancy, parity, pre-pregnancy BMI, season at delivery, income, asthma, thyroid disease, malignant tumors, liver disease, and hypertension or diabetes.

$^{+}$ p for interaction < 0.1. *p for interaction < 0.05.

Table S4. Associations between air pollution exposure (per IQR increase) and PSS scores after excluding women with a chronic health condition

Air pollutants	Trimester	β (95% CI)
$PM_{2.5}$ (IQR: 6.88 μg/m^3)	First	0.37 (0.07, 0.67) *
	Second	0.43 (0.12, 0.74) **
	Third	−0.07 (−0.36, 0.23)
	Pregnancy	0.43 (0.03, 0.82) *
PM_{10} (IQR: 8.86 μg/m^3)	First	0.35 (0.09, 0.61) **
	Second	0.42 (0.13, 0.71) **
	Third	−0.17 (−0.43, 0.10)
	Pregnancy	0.60 (0.14, 1.07) *
NO_2 (IQR: 9.0 ppb)	First	0.16 (−0.14, 0.47)
	Second	0.07 (−0.27, 0.42)
	Third	−0.27 (−0.57, 0.04) $^{+}$
	Pregnancy	−0.003 (−0.37, 0.36)
O_3 (IQR: 11.0 ppb)	First	−0.17 (−0.42, 0.07)
	Second	0.29 (−0.02, 0.61) $^{+}$
	Third	0.38 (0.12, 0.63) **
	Pregnancy	0.46 (−0.04, 0.96) $^{+}$

n = 1854. IQR, interquartile range; CI, confidence interval. Models adjusted for maternal age, education, occupation, family income, gestational age, maternal smoking, drinking during pregnancy, pre-pregnancy BMI, parity, and season at delivery.

$^{+}$ p-value < 0.1. *p-value < 0.05. **p-value < 0.01.

Table S5. Comparison of imputation methods using adjusted models

Air pollutants	Trimester	Simple mean substitution	Multiple imputation[a]
		β (95% CI)	β (95% CI)
$PM_{2.5}$ (IQR: 6.88 μg/m^3)	First	0.29 (0.02, 0.56) *	0.30 (0.02, 0.57) *
	Second	0.36 (0.07, 0.65) *	0.37 (0.08, 0.65) *
	Third	0.01 (−0.26, 0.28)	0.01 (−0.26, 0.28)
	Pregnancy	0.39 (0.03, 0.74) *	0.39 (0.03, 0.75) *
PM_{10} (IQR: 8.86 μg/m^3)	First	0.26 (0.02, 0.50) *	0.27 (0.03, 0.51) *
	Second	0.34 (0.08, 0.61) *	0.35 (0.08, 0.61) *
	Third	−0.08 (−0.32, 0.16)	−0.08 (−0.31, 0.16)
	Pregnancy	0.53 (0.11, 0.95) *	0.54 (0.11, 0.96) *
NO_2 (IQR: 9.0 ppb)	First	0.14 (−0.13, 0.41)	0.14 (−0.13, 0.41)
	Second	0.11 (−0.20, 0.43)	0.11 (−0.20, 0.42)
	Third	−0.17 (0.45, 0.11)	−0.17 (−0.45, 0.11)
	Pregnancy	0.04 (−0.29, 0.38)	0.04 (−0.29, 0.38)
O_3 (IQR: 11.0 ppb)	First	−0.14 (−0.36, 0.09)	−0.14 (−0.36, 0.08)
	Second	1.13 (−0.15, 0.42)	0.14 (−0.15, 0.42)
	Third	0.25 (0.02, 0.48) *	0.25 (0.02, 0.48) *
	Pregnancy	0.22 (−0.22, 0.67)	0.22 (−0.22, 0.67)

n = 2227. IQR, interquartile range; CI, confidence interval. The model 4 shown in Table 3 was used for th e analysis.

[a] Missing data was filled in using multiple imputation technique following a Markov Chain Monte Carlo distribution.

*p-value < 0.05.

Table S6. Sensitivity analysis using E-value estimates

Air pollutant	Trimester	RR (95% CI)[a]	E-value[b]
$PM_{2.5}$ (IQR = 6.88 μg/m^3)	First	1.014 (1.005, 1.023) **	1.13 (1.08)
	Second	1.018 (1.008, 1.027) ***	1.15 (1.10)
	Third	0.998 (0.990, 1.007)	1.05 (1.00)
	Pregnancy	1.018 (1.006, 1.030) **	1.15 (1.08)
PM_{10} (IQR = 8.86 μg/m^3)	First	1.014 (1.006, 1.022) ***	1.13 (1.08)
	Second	1.018 (1.009, 1.027) ***	1.15 (1.10)
	Third	0.995 (0.987, 1.003)	1.08 (1.00)
	Pregnancy	1.027 (1.013, 1.041) ***	1.19 (1.13)
NO_2 (IQR = 9.0 ppb)	First	1.009 (0.999, 1.018) +	1.10 (1.00)
	Second	1.006 (0.996, 1.017)	1.08 (1.00)
	Third	0.992 (0.983, 1.001)	1.10 (1.00)
	Pregnancy	1.003 (0.992, 1.014)	1.06 (1.00)
O_3 (IQR = 11.0 ppb)	First	0.993 (0.986, 1.0001) +	1.09 (1.00)
	Second	1.009 (1.00004, 1.019) *	1.10 (1.01)
	Third	1.015 (1.007, 1.023) ***	1.14 (1.09)
	Pregnancy	1.016 (1.001, 1.031) *	1.14 (1.03)

n = 2153. IQR, interquartile range; CI, confidence interval.

[a] The relative risk (RR) and 95% CIs are obtained from Poisson regression model using the model 4 shown in Table 3, indicating RR of perceived stress for interquartile range change in air pollution exposure during pregnancy.

[b] E-values for point estimates (RR) and corresponding CIs. Lower 95% CIs are reported in parentheses.

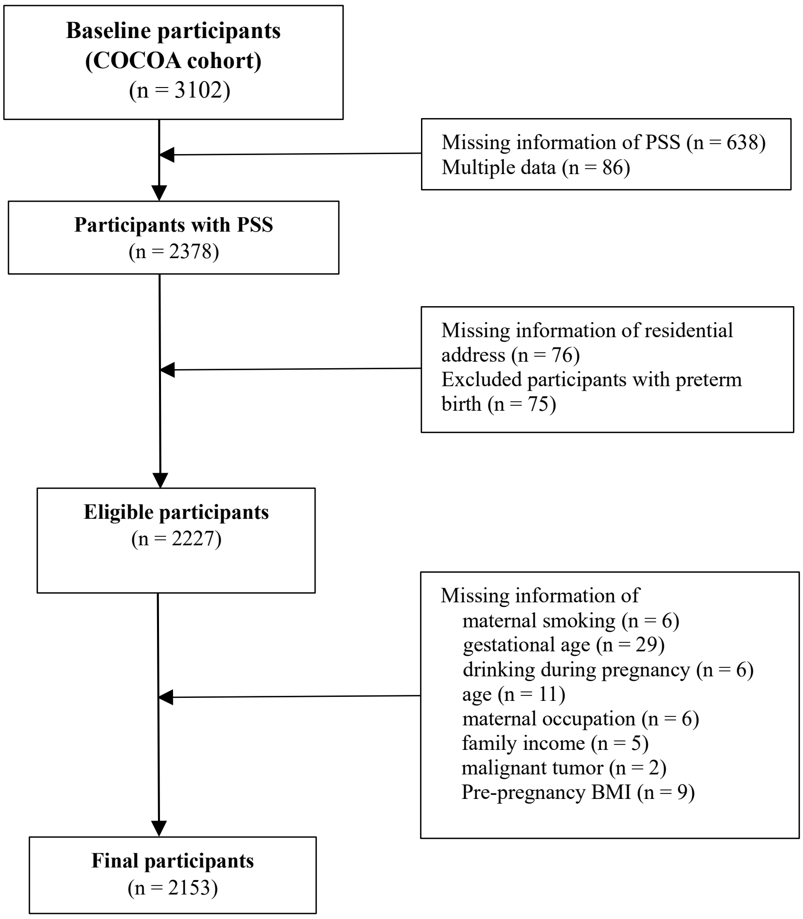

Figure S3. Final analytic sample and sample size. The figure shows how the final analytic sample used in this study was derived from the Cohort for Childhood Origin of Asthma and allergic diseases (COCOA). It shows the application of exclusion criteria based on exposure and outcome variables of interest and availability of critical variables for modeling. BMI, body mass index; PSS, perceived stress scale.

Table S7. Characteristics of the study participants from the COCOA cohort included and not included in the present study

Characteristics	Included (n = 2153)	Excluded (n = 74)	p-value
**Age (years)**	33.1 ± 3.6	32.8 ± 3.5	0.510
**Pre-pregnancy BMI (kg/m^2)**			
< 25	2012 (93.5)	61 (82.4)	0.118
≥ 25	141 (6.5)	1 (1.4)	
**Parity**			
Nulliparous	1314 (61.0)	38 (51.4)	0.555
Parous	661 (30.7)	16 (21.6)	
**History of smoking**			
Never	1988 (92.3)	61 (82.4)	0.424
Ever	165 (7.7)	7 (9.5)	
**Drinking during pregnancy**			
No	1989 (92.4)	61 (82.4)	0.685
Yes	164 (7.6)	6 (8.1)	
**Occupation**			
No	724 (33.6)	28 (37.8)	0.137
Yes	1429 (66.4)	38 (51.4)	
**Education**			
Secondary school	99 (4.6)	4 (5.4)	0.788
College or university	1579 (73.3)	56 (75.7)	
Graduate school	475 (22.1)	14 (18.9)	
**Gestational age (weeks)**	39.3 ± 1.1	39.2 (1.0)	0.514
**Family income**			
High (≥ 4 million per month)	1353 (62.8)	34 (45.9)	0.031
Low (< 4 million per month)	800 (37.2)	34 (45.9)	
**Asthma**			
No	2081 (96.7)	73 (98.6)	0.344
Yes	72 (3.3)	1 (1.4)	
**Thyroid disease**			
No	2011 (93.4)	70 (94.6)	0.684
Yes	142 (6.6)	4 (5.4)	
**Malignant tumor**			
No	2123 (98.6)	71 (95.9)	0.997
Yes	30 (1.4)	1 (1.4)	
**Liver disease**			
No	2084 (96.8)	72 (97.3)	0.809
Yes	69 (3.2)	2 (2.7)	
**Hypertension or Diabetes mellitus**			
No	2121 (98.5)	73 (98.6)	0.925
Yes	32 (1.5)	1 (1.4)	
**Season at delivery**			
Spring	465 (21.6)	7 (9.5)	0.089
Summer	432 (20.1)	18 (24.3)	
Autumn	489 (22.7)	15 (20.3)	
Winter	590 (27.4)	13 (17.6)	

*p values obtained from chi square test for categorical variables and t-test for continuous variables. Data are shown as n (%) or mean ± standard deviation. Of the 225 participants with PSS who were excluded, we considered 74 after removing 151 participants with missing information of residential addresses and preterm birth. Total numbers may not be equal to the total included and excluded numbers for some characteristics due to missing data. BMI, body mass index.
